# The Calcium-Dependent Protein Kinase *TaCDPK27* Positively Regulates Salt Tolerance in Wheat

**DOI:** 10.3390/ijms23137341

**Published:** 2022-07-01

**Authors:** Jie-Yu Yue, Jin-Lan Jiao, Wen-Wen Wang, Hua-Zhong Wang

**Affiliations:** Tianjin Key Laboratory of Animal and Plant Resistance, College of Life Sciences, Tianjin Normal University, Tianjin 300387, China; skyyjy@tjnu.edu.cn (J.-Y.Y.); 13834612344@163.com (J.-L.J.); wangww0630@163.com (W.-W.W.)

**Keywords:** calcium-dependent protein kinase, *TaCDPK27*, NaCl stress, PCD, wheat seedling

## Abstract

As essential calcium ion (Ca^2+^) sensors in plants, calcium-dependent protein kinases (CDPKs) function in regulating the environmental adaptation of plants. However, the response mechanism of CDPKs to salt stress is not well understood. In the current study, the wheat salt-responsive gene *TaCDPK27* was identified. The open reading frame (ORF) of *TaCDPK27* was 1875 bp, coding 624 amino acids. The predicted molecular weight and isoelectric point were 68.905 kDa and 5.6, respectively. *TaCDPK27* has the closest relationship with subgroup III members of the CDPK family of rice. Increased expression of *TaCDPK27* in wheat seedling roots and leaves was triggered by 150 mM NaCl treatment. TaCDPK27 was mainly located in the cytoplasm. After NaCl treatment, some of this protein was transferred to the membrane. The inhibitory effect of *TaCDPK27* silencing on the growth of wheat seedlings was slight. After exposure to 150 mM NaCl for 6 days, the NaCl stress tolerance of *TaCDPK27*-silenced wheat seedlings was reduced, with shorter lengths of both roots and leaves compared with those of the control seedlings. Moreover, silencing of *TaCDPK27* further promoted the generation of reactive oxygen species (ROS); reduced the activities of superoxide dismutase (SOD), peroxidase (POD) and catalase (CAT); aggravated the injury to photosystem II (PS II); and increased programmed cell death (PCD) in wheat leaves under NaCl treatment, confirming that the *TaCDPK27*-silenced seedlings exhibited more NaCl injury than control seedlings. Taken together, the decrease in NaCl tolerance in *TaCDPK27*-silenced seedlings was due to excessive ROS accumulation and subsequent aggravation of the NaCl-induced PCD. *TaCDPK27* may be essential for positively regulating salt tolerance in wheat seedlings.

## 1. Introduction

Plant growth is affected by various environmental stresses (such as, salinity, drought, cold, etc.) throughout the plant life cycle, reducing growth and productivity [1,2,3]. To survive these stresses, plants have developed multi-level defense strategies that guarantee normal growth and productivity [4,5,6]. Various proteinaceous and non-proteinaceous elements are essential in these plant defense responses [3]. Proteinaceous factors with this role include enzymes, transcription factors, and receptors, and such non-proteinaceous elements include a number of secondary messengers, such as calcium ions (Ca^2+^), cyclic nucleotides, hydrogen ion (H^+^), lipids, and reactive oxygen species (ROS) [7,8,9,10,11]. Among them, Ca^2+^ is a vital secondary messenger that functions in signal transduction in plant growth and development [12]. Upon stimulation by the external environment, plants experience concentration fluctuation of the cytoplasmic Ca^2+^ called Ca^2+^ oscillation, which is perceived signals to trigger downstream responses [13]. These Ca^2+^ signals activate calcium-sensing proteins, thereby regulating the expression of defense genes and activating the distinct physiological and biochemical response pathways of plants [14]. Plants have four types of calcium-sensing proteins, including calmodulin (CaM), CaM-like protein (CML), calcineurin B-like protein (CBL), and calcium-dependent protein kinase (CDPK) [15]. As for the CDPKs we were concerned with in this study, plant CDPKs have four functional domains consisting of a highly variable N-terminal domain (dual leucine-zipper kinase, DLK) motif, serine (Ser)/threonine (Thr) kinase catalytic domain (S-TKc), autoinhibitory junction domain, and CaM-like domain [16,17]. In addition, the four EF-hand motifs distribute in the N-termini of CDPK sequences and are responsible for protein activation via binding Ca^2+^ [16,17].

Growing evidence has confirmed the important roles of plant CDPKs in stress signal transduction [16]. Barley *HvCPK2a* and wheat *TaCDPK34* are CDPKs proved to be involved in the plant response to drought stress with opposite roles [18,19]. Overexpression of *HvCPK2a* led to increased drought sensitivity in barley [18], while it is silencing of *TaCDPK34* that leads to a similar phenotype in wheat [19]. Rice *OsCDPK21* and *OsCDPK10*, *Glycyrrhiza uralensis GuCDPKs*, maize *ZmCDPK11*, and apple *MdCDPK1a* are CDPKs improved by plant responses to salt stress [17,20,21,22,23]. *OsCDPK21* confer salt tolerance by modulating the abscisic acid (ABA) signal pathway and phosphorylating *OsGF14e*, a 14-3-3 protein [21]. *OsCPK10* and *MdCDPK1a* achieved this by positively regulating the cellular anti-oxidative capacity [20,23]. *OsCDPK12*, another rice CDPK, also plays an antioxidation role to protect plants from early leaf senescence [24]. *GuCPKs* contribute salt tolerance of *G. Uralensis* plants by increasing glycyrrhizic acid and flavonoid synthesis [16]. *MdCDPK1a* of apple also functions in plant cold tolerance, and a similar role is played by another rice CDPK, *OsCPK24*, which can inhibit the glutaredoxin *OsGrx10* to maintain high levels of glutathione [23,25]. *ZmCDPK7*, another maize CDPK, positively regulates maize tolerance to heat stress [8]. In addition to the roles of CDPKs in plant responses to abiotic stresses, roles of plant CDPKs in plant responses to biotic stressed have also been reported. *AtCDPK28*, an *Arabidopsis* CDPK, inhibits the *BIK1*-mediated immune response by phosphorylating *BIK1* for turnover [26]. The above-mentioned rice *OsCDPK10* and another wheat CDPK, *TaCDPK7-D*, however, function as positive regulators, respectively, in rice resistance to blast disease and in wheat resistance to *Rhizoctonia cerealis* infection with the assistance of several defense-related genes and *ACO2* controlling ethylene biosynthesis [20,27].

Plant CDPK is a multi-protein family with 34 members in *Arabidopsis thaliana* [28], 31 CDPKs in rice [21], 30 CDPKs in poplar [29], 41 CDPKs in cotton [30], and 20 CDPKs in bread wheat [19]. To date, only a small number of plant CDPKs have been functionally characterized. The common wheat (*Triticum aestivum* L.) is a major food crop worldwide that provides dietary carbohydrates for more than one-third of the world’s population [31]. In this study, we report the characterization of a new wheat CDPK gene, *TaCDPK27-5B*, and provide evidence to show that *TaCDPK27-5B* is engaged in wheat response to salt stress.

## 2. Results

### 2.1. NaCl Treatment Increases Ca^2+^ Levels in the Roots of Wheat Seedlings

**Figure 1:**
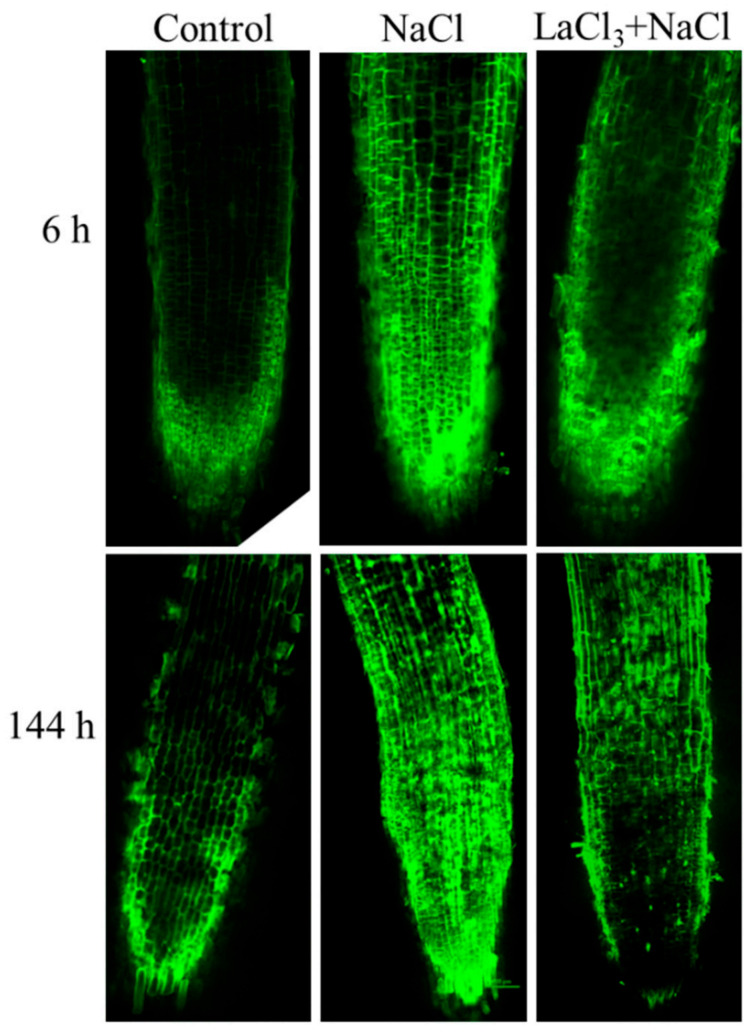
Confocal images of Fluo-3/AM fluorescence showing Ca^2+^ accumulation in roots of wheat seedlings within NaCl treatment with or without LaCl_3_ pretreatment.

To detect whether the Ca^2+^ signal is involved in the wheat response to NaCl treatment, the fluorescence dye Fluo-3/AM was used to measure the level of intracellular Ca^2+^ in the roots of wheat seedlings subjected to NaCl treatment. The fluorescence intensity of Ca^2+^-bound Fluo-3 positively associates with the level of Ca^2+^ in the cell. A confocal laser scanning microscope showed that 6 h and 144 h treatment with 150 mM NaCl resulted in intensively enhanced Fluo-3 fluorescence in seedling roots suggesting an elevated level of Ca^2+^.This NaCl-induced elevation of the Ca^2+^ level was compromised by application of the Ca^2+^-channel blocker La^3+^ (Figure 1). These results suggest that the second messenger Ca^2+^ in seeding roots responds to NaCl stress by migrating into cells.

### 2.2. Cloning and Characterization of the Wheat CDPK Gene TaCDPK27B

**Figure 2:**
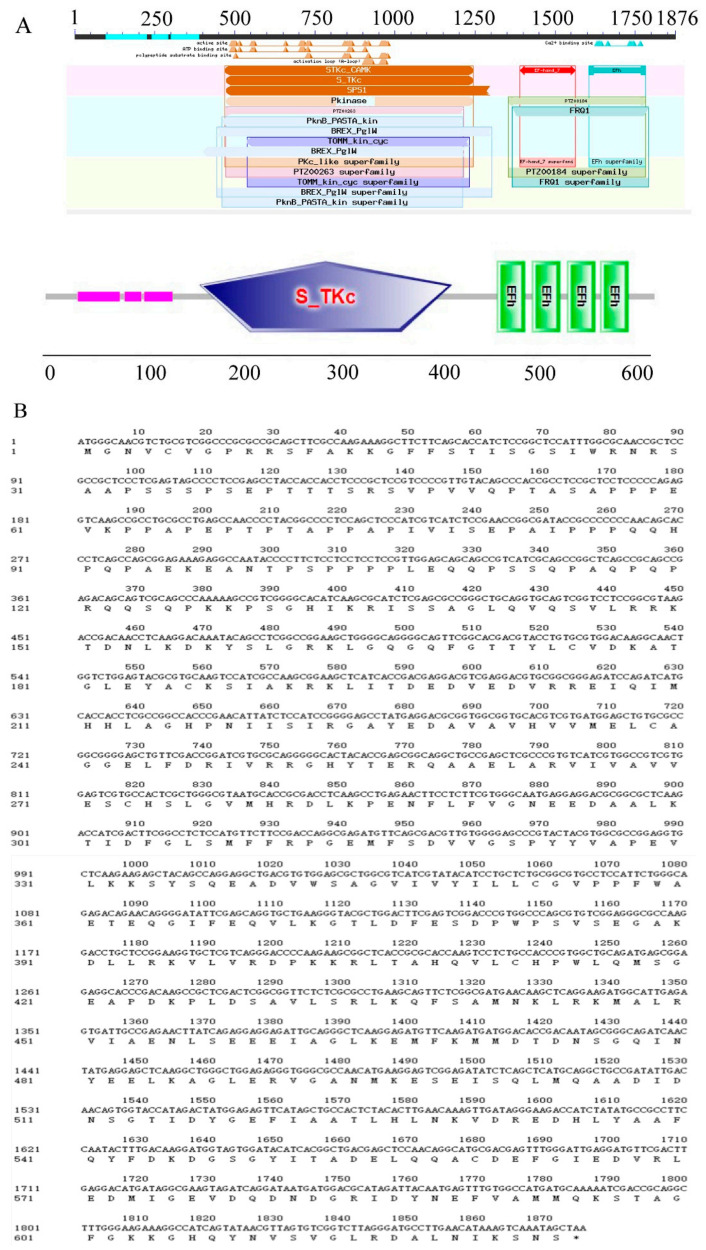
Conserved domain analysis of a calcium-dependent protein kinase gene, *TaCDPK27*, in wheat. (**A**) Domain structure of TaCDPK27. TaCDPK27 protein contained an N-terminal variable domain, a serine/threonine kinase-like domain (S-TKc), and four EF-hand motifs. (**B**) Nucleotide and amino acid sequences of TaCDPK27.

**Figure 3:**
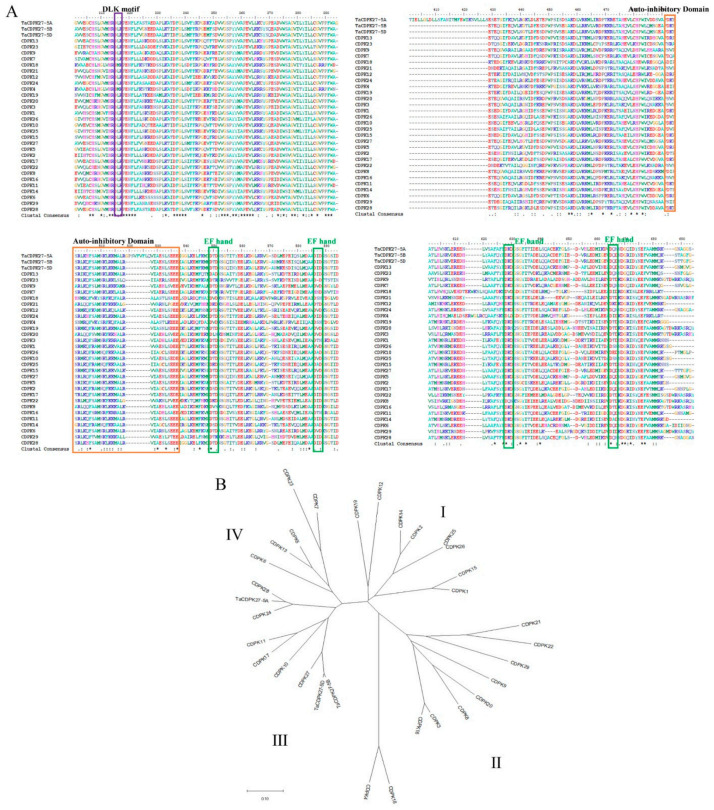
Sequence analysis of TaCDPK27. (**A**) Multi-sequence alignment of TaCDPK27 with other CDPK proteins from rice. Amino acid sequences from 29 function-known CDPKs share similar structures, containing DLK motif, a serine/threonine kinase-like domain (S-TKc), and four EF-hand motifs. Ta, *Triticum aestivum* L. (**B**) Phylogenetic analysis of TaCDPK27. I, Ⅱ, III and IV indicate the subgroups of CDPK family. TaCDPK27 and function-known CDPKs in rice are used to construct their phylogenetic tree using MEGA-X software.

After amplification, the complete coding sequence (CDS) of the salt-responsive unigene (accession no. TraesCS5B02G109300) was acquired. Sequence analysis showed that the whole length of the open reading frame (ORF) was 1875 bp, which encoded 624 amino acids with a molecular weight and theoretical isoelectric point (pI) of 68.905 kDa and 5.6, respectively. The protein had the typical characteristics of CDPKs, an N-terminal variable domain; S-TKc, an autoinhibitory domain; and four EF-hand motifs that could activate proteins by binding Ca^2+^ (Figure 2). The deduced amino acid alignment analysis indicated 99% similarity to CDPK27-like isoform X in both Triticum dicoccoides and Aegilops tauschii subsp. strangulata, and it was located on chromosome 5B, so we named it TaCDPK27B (TaCDPK27). According to Chen et al. (2020) [32], TaCDPK2B was used as a probe to search for homologs in the Triticeae-GeneTribe database. TraesCS5D02G124000, located on chromosome 5D, and TraesCS5A02G107900, located on chromosome 5A, were identified as TaCDPK27B homologs in wheat and designated TaCDPK27D and TaCDPK27A, respectively. The ORF nucleotide sequences of TaCDPK27A, TaCDPK27B, and TaCDPK27D share identities of 53–98% and encode peptide sequences of 564, 624, and 624 amino acids, respectively. Phylogenetic analysis showed that the TaCDPK27-5A protein shared high similarities with OsCDPK24 and OsCDPK28. Both TaCDPK27-5B and TaCDPK27-5D shared high similarities with OsCDPK27. The three TaCDPKs were all in subgroup III of the CDPK family (Figure 3).

### 2.3. TaCDPK27 Responds to NaCl Treatment

**Figure 4:**
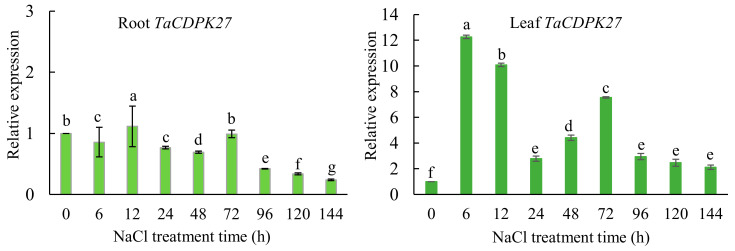
Transcript levels of *TaCDPK27* in roots and leaves of wheat seedlings under NaCl treatment determined using qRT-PCR assay. Data were presented as mean ± SD from at least three independent experiments. Bars with different letters are significantly different at *p* < 0.05.

Quantitative real-time PCR (qRT-PCR) assays were conducted to investigate the expression response of *TaCDPK27* to a time-series NaCl treatment in wheat seedlings. As shown in Figure 4, the expression of *TaCDPK27* was significantly unregulated in leaves within 72 h of NaCl treatment. In the roots of NaCl-treated seedlings, this expression upregulation of *TaCDPK27* within 72 h of NaCl treatment was negligible, and significantly decreased expression of *TaCDPK27B* was detected in the roots after 72 h of NaCl treatment.

**Figure 5:**
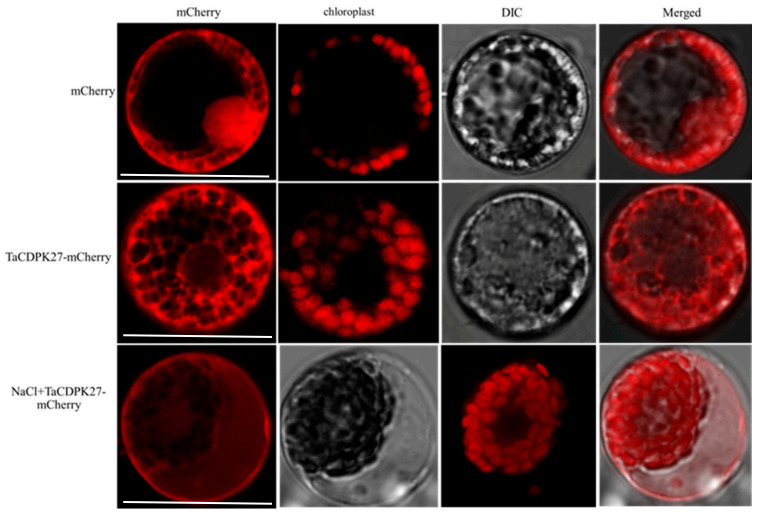
Subcellular localization of TaCDPK27. Wheat protoplasts were transformed with *pAN583:mCherry* or *TaCDPK27-mCherry* via the polyethylene glycol (PEG)-mediated method. TaCDPK27 is mainly localized to the cytoplasm in wheat protoplasts. Under salt stress conditions, some of TaCDPK27 was transferred to the membrane. Bar = 50 μm.

Plant CDPKs have been shown to localize in different cellular compartments implying their functional diversification [33,34] Here, mCherry and TaCDPK27-mCherry were each expressed in wheat mesophyll protoplasts to investigate the localization of TaCDPK27 (Figure 5). Under normal conditions for the maintenance of transfected protoplasts, the fluorescence signals of mCherry spread in the whole cell including the nucleus, while those of TaCDPK27-mCherry only spread in the cytoplasm. Interestingly, a 150 mM NaCl treatment to the protoplasts resulted in translocation of a majority of TaCDPK27-mCherry signals to the periphery of the cell (Figure 5). These results suggest that wheat seedling leaf TaCDPK27 responds to NaCl treatment by upregulation of the transcription of *TaCDPK27* and the translocation of TaCDPK27.

### 2.4. Silencing of TaCDPK27 Increases the Salt Sensitivity of Wheat Seedlings

**Figure 6:**
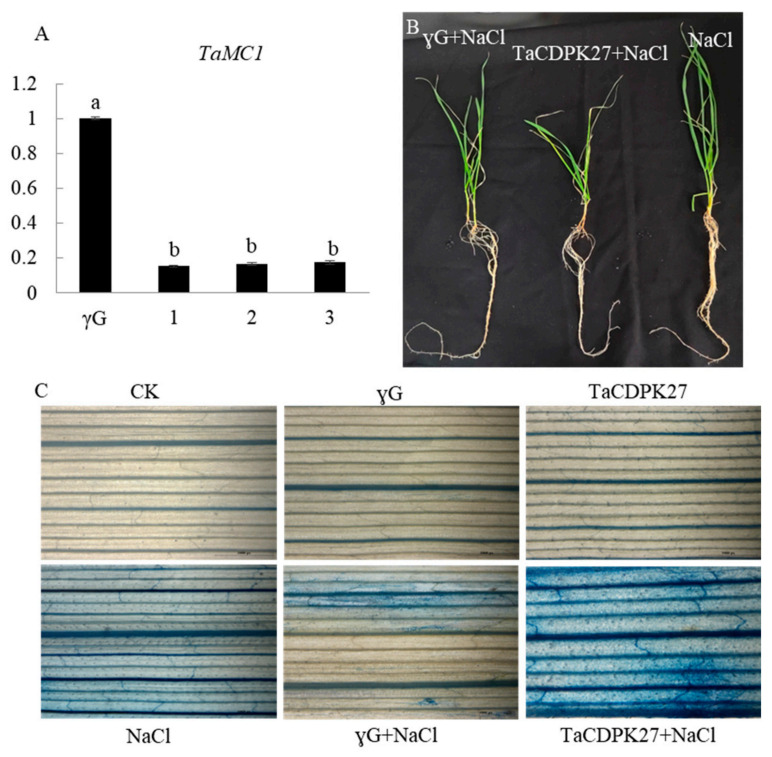
The effect of silencing *TaCDPK27* on the cell death in leaves of wheat seedlings under NaCl stress. (**A**) Relative transcript levels of *TaCDPK27* in leaves of BSMV-inoculated wheat seedlings. Standard deviations are shown (*n* > 3, ±SD, *p* < 0.05). CK was from wild-type wheat seedlings, γG was from BSMV-VIGS-GFP-inoculated wheat seedlings, *TaCDPK27* was from BSMV-VIGS-TaCDPK27-inoculated wheat seedlings, and arabic numbers indicate individual *TaCDPK27*-silenced seedling. The data with different letters (a & b) in same column show significant difference (*p* < 0.05). (**B**) The effect of TaCDPK27 silencing on growth of wheat seedlings under NaCl treatment. (**C**) Evans blue staining results.

We then sought to get clear information on the role of *TaCDPK27* in wheat response to NaCl stress through gene silencing. *TaCDPK27*-silenced seedlings were prepared by inoculating with the method of the barley stripe mosaic virus (BSMV)-based virus-induced gene silencing (VIGS). To further confirm the roles of *TaCDPK27* in the salt stress response of wheat seedlings, *TaCDPK27*-silenced wheat seedlings were acquired using BMSV-VIGS. After 6 days of BMSV inoculation, the third leaves of BSMV-VIGS-inoculated seedlings displayed chlorosis, confirming that the VIGS system functioned correctly. Then qRT-PCR was employed to determine the expression level of *TaCDPK27*. Compared with the control, the expression of *TaCDPK27* was significantly inhibited in the leaves of *TaCDPK27*-silenced seedlings (Figure 6A).

To analyze the effect of *TaCDPK27* silencing on wheat seedling tolerance to salt stress, *TaCDPK27*-silenced seedlings were treated with 150 mM NaCl for 6 days. Silencing of *TaCDPK27* slightly inhibited increases in root and leaf length (Table 1). After exposure to NaCl treatment for 6 days, silencing of *TaCDPK27* aggravated the NaCl stress-induced damage to wheat seedling growth (Figure 6B), and the root length of *TaCDPK27*-silenced seedlings was decreased by 28.34% (8.73 cm), compared with 19.63% (6.3 cm) for BSMV-GFP-inoculated-seedlings and 20.55% (7.5 cm) for the wild-type seedlings. Likewise, the leaf length of *TaCDPK27*-silenced seedlings was decreased by 17.97% (4.9 cm), compared with 16.76% (4.9 cm) for BSMV-GFP-inoculated-seedlings and 16.50% (5.13 cm) for wild-type seedlings under NaCl treatment (Table 1). Evans blue staining also showed that silencing *TaCDPK27* aggravated NaCl stress-induced cell death in the leaves of wheat seedlings (Figure 6C). The above results suggested that silencing *TaCDPK27* increased wheat seedling sensitivity to NaCl treatment.

### 2.5. Silencing of TaCDPK27 Promoted NaCl Stress-Triggered Programmed Cell Death (PCD) in Leaves of Wheat Seedlings

**Figure 7:**
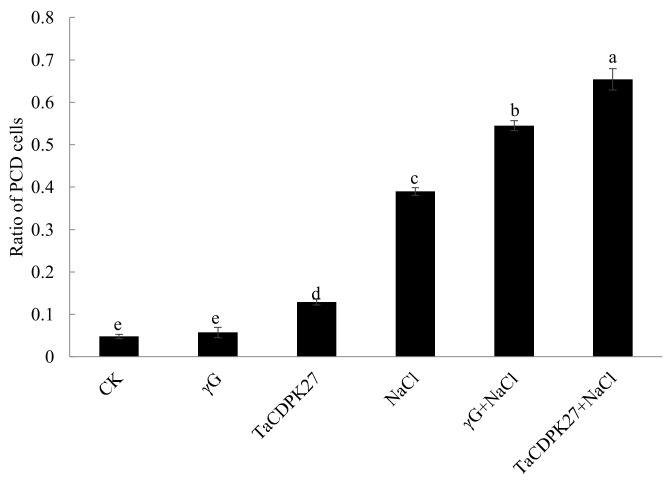
The effect of silencing *TaCDPK27* on PCD level in leaves of wheat seedlings under NaCl treatment. CK was from wild-type wheat seedlings, γG was from BSMV-VIGS-GFP-inoculated wheat seedlings, and TaCDPK27 was from BSMV-VIGS-TaCDPK27-inoculated wheat seedlings. Bars with different letters are significantly different at *p* < 0.05.

To investigate the potential role of *TaCDPK27* in PCD, a TdT-mediated dUTP Nick-End Labeling (TUNEL) assay was used to detect the PCD level in leaves of wheat seedlings. The results showed that NaCl treatment induced PCD with increased TUNEL-positive nuclei in wheat leaves at the seedling stage. Silencing *TaCDPK27* further increased NaCl stress-triggered TUNEL-positive nuclei (Figure 7). These results revealed that *TaCDPK27* regulated the PCD triggered by NaCl treatment in wheat leaves at the seedling stage.

### 2.6. Silencing TaCDPK27 Aggravates the NaCl Stress-Induced Injury of Photosystem II (PSII)

Compared with the control, NaCl stress significantly decreased PSII photochemistry (Fv/Fm), quantum yield of PSII (Y(II)), quenching coefficient (qP), minimal fluorescence (F_0_), and electron transfer rate (ETR) and increased nonphotochemical quenching coefficient (NPQ) in wheat seedlings. After exposure to NaCl stress for 6 days, the Fv/Fm of *TaCDPK27*-silenced seedlings was decreased by 8.47%, compared with 6.47% for BSMV-GFP-inoculated seedlings and 4.78% for wild-type seedlings. After exposure to NaCl stress for 6 days, the Y(II) of *TaCDPK27*-silenced seedlings was decreased by 46.67%, compared with 35.80% for BSMV-GFP-inoculated seedlings and 23.93% for wild-type seedlings. After exposure to NaCl stress for 6 days, the qP of *TaCDPK27*-silenced seedlings was decreased by 38.87%, compared with 24.66% for BSMV-GFP-inoculated seedlings and 12.38% for wild-type seedlings. After exposure to NaCl stress for 6 days, the F0 of *TaCDPK27*-silenced seedlings was decreased by 43.86%, compared with 36.51% for BSMV-GFP-inoculated seedlings and 33.33% for wild-type seedlings. After exposure to NaCl stress for 6 days, the ETR of *TaCDPK27*-silenced seedlings was decreased by 46.03%, compared with 35.88% for BSMV-GFP-inoculated seedlings and 22.57% for wild-type seedlings. After exposure to NaCl stress for 6 days, the NPQ of *TaCDPK27*-silenced seedlings was increased by 110.24%, compared with 95.36% for BSMV-GFP-inoculated seedlings and 123.85% for wild-type seedlings (Table 2). These results showed that silencing of TaCDPK27 further decreased Fv/Fm, Y(II), qP, F_0_, and ETR and increased NPQ in wheat seedlings under NaCl treatment, confirming that knockdown of *TaCDPK27* aggravated NaCl treatment induced PSII injury in wheat seedlings.

### 2.7. Silencing TaCDPK27 Reduces the Antioxidant Capacity of Wheat Roots and Leaves under NaCl Treatment

**Figure 8:**
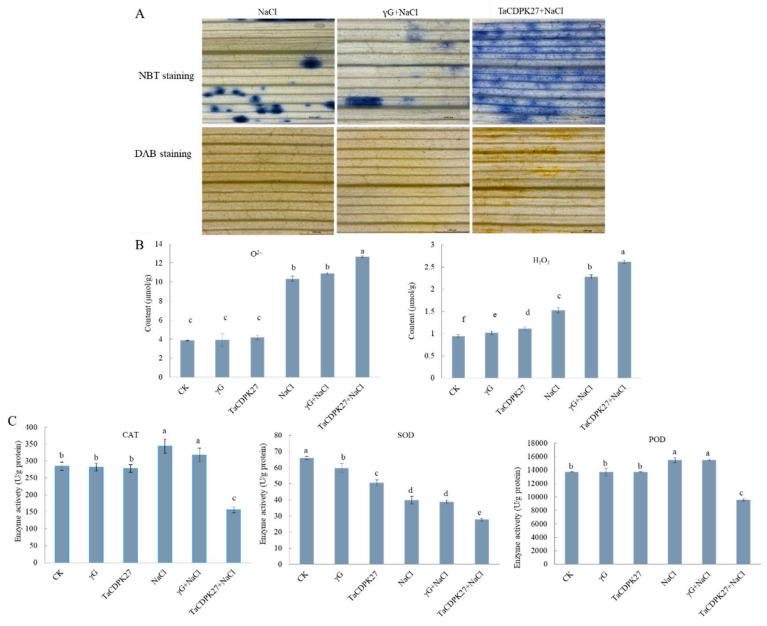
The effect of silencing of *TaCDPK27* on the ROS accumulation in leaves of wheat seedlings under NaCl treatment. (**A**) NBT staining of the generation and accumulation of O^2−.^ in leaves of wheat seedlings. DAB staining of the generation and accumulation of H_2_O_2_ in leaves of wheat seedlings. (**B**) The content of O^2−.^ and H_2_O_2_ in leaves of wheat seedlings. (**C**) The activities of POD, SOD, and CAT in leaves of wheat seedlings. Standard deviations are shown (*n* > 3, ±SD, *p* < 0.05). CK was from wild-type wheat seedlings, γG was from BSMV-VIGS-GFP-inoculated wheat seedlings, TaCDPK27 was from BSMV-VIGS-TaCDPK27-inoculated wheat seedlings. The different letters in each treatment show the significant difference (*p* < 0.05). All the experiments presented here were performed at least three times, and similar results were obtained.

To study the influence of *TaCDPK27* on the antioxidant capacity of wheat leaves under NaCl treatment, the ROS levels and the activities of some antioxidant enzymes were monitored. 3,3-diaminobenzidine (DAB) and nitro blue tetrazolium (NBT) staining methods were employed to determine the generation of O^2−.^ and H_2_O_2_ in the wheat leaves, respectively. Under normal growth conditions, silencing of *TaCDPK27* had no obvious effect on the accumulation of O^2−.^ and had a little effect on the accumulation of H_2_O_2_ in the leaves of wheat seedlings. NaCl treatment increased excess O^2−.^ and H_2_O_2_ in the leaves of wheat seedlings. Silencing of *TaCDPK27* further promoted the generation of O^2−.^ and H_2_O_2_ under NaCl treatment (Figure 8A). Quantitative detection of O^2−.^ and H_2_O_2_ were consistent with the NBT and DAB staining results, respectively (Figure 8B). The activities of superoxide dismutase (SOD), peroxidase (POD), and catalase (CAT) in the leaves of wheat seedlings were markedly increased under NaCl treatment compared with those of the control (Figure 8C). The *TaCDPK27*-silenced seedlings had significantly lower SOD, POD, and CAT activities than γG and WT seedlings after exposure to NaCl treatment. These results suggested that silencing *TaCDPK27* reduced the antioxidant capacity of wheat leaves under NaCl treatment.

## 3. Discussion

Salt stress has become one of the main factors threatens to plant growth and crop production [35]. Plants adapt to salt stress through the activation of many physiological and metabolic responses, due to the perception of stress signals and subsequent signal transduction [36,37]. As a secondary messenger, Ca^2+^ functions in the plant response to environmental stresses by coupling extracellular signals with intracellular physiological and biochemical responses with Ca^2+^ sensors or Ca^2+^-binding proteins [12,13]. CDPKs are widespread calcium receptors in protists, oomycetes, green algae, and plants that form a large multigene family and are separated into four types (I, II, III, and IV) [23,27,38]. Genome research has showed that 34 *CDPKs* in *Arabidopsis* [28], 31 *CDPKs* in rice [21], 20 *CDPKs* in bread wheat [19], 30 *CDPKs* in poplar [29], and 41 *CDPKs* in cotton [30] have been identified. They have biological control functions in plant responses to various environmental stresses [38]. In the present study, the Ca^2+^ signal in wheat roots and leaves was induced significantly under NaCl stress. A similar phenomenon was observed in *Arabidopsis* induced by Ma et al. (2019) [39]. The *TaCDPK27* was also identified as a salt-responsive gene, which possessed conserved domains similar to those of CDPKs, including an N-terminal variable domain, an S-TKc, an autoinhibitory domain, and four Ca^2+^-binding EF-hand motifs. TaCDPK27 has the closest relationship with subgroup III members of the CDPK family of rice. Unlike AtCPK27, which is a membrane-localized protein kinase [40], TaCDPK27 was localized to the cytoplasm, and the localization could be changed to the membrane under NaCl stress, indicating that *TaCDPK27* might be functional in multiple signal transduction pathways. Our results suggested that the *TaCDPK27*-mediated Ca^2+^ signal was involved in the regulation of wheat salt tolerance, which is consistent with research on licorice, *Arabidopsis*, rice, and maize [17].

Studies have explored evidence that CDPKs are important regulators of the plant defense response to multiple stress. *CDRK2* reduced the salt sensitivity of *Arabidopsis* [41]. *AtCDPK27* was required for *Arabidopsis* adaptation to salt stress by regulating ion and ROS homeostasis [40]. Overexpression of *VaCDPK20* elevated the tolerance of *Arabidopsis* under freezing and drought treatment conditions [42]. *ZmCDPK7* conferred maize tolerance to heat stress, which was induced by ROS accumulation [8]. *TaCDPK27* is also involved in the wheat response to NaCl treatment at the seedling stage. Silencing of *TaCDPK27* enhanced the sensitivity of wheat seedlings to NaCl treatment, whereas under normal growth conditions, it showed a slight effect on plant growth. Moreover, silencing of *TaCDPK27* decreased SOD, POD, and CAT activities in the leaves of wheat seedlings treated with NaCl stress, while the silencing of *TaCDPK27* led to increase of O^2−.^ and H_2_O_2_ induced by NaCl treatment. Consequently, the deterioration of PSII became more serious. Our results align with those of the previous study that overexpression of *OsCDPK12* induces delayed leaf senescence by reducing oxidative damage and enhancing the net photosynthesis rate (Pn) and chlorophyll content in rice [24]. These results indicated that silencing of *TaCDPK27* aggravated NaCl stress-induced damage to the photosynthetic system by decreasing SOD, POD, and CAT activities to increase ROS generation in NaCl-treated wheat seedlings.

PCD can eliminate unwanted cells in the plant defense response [43]. However, the signaling pathways underlying the triggering of PCD remain unclear. Previous studies have proposed that excess ROS can induce PCD in plants [44]. The current results indicated that *TaCDPK27* is involved in NaCl stress-induced PCD. Silencing of *TaCDPK27* caused an increase in both O^2−^ and H_2_O_2_ contents and a higher level of PCD induced by NaCl treatment. Our results indicated that *TaCPK27* positively regulates ROS-scavenging in wheat seedlings, which benefits adaptation to NaCl stress. Connections among *TaCDPK27*, ROS homeostasis, and PCD have long been established. *TaCDPK27* is mainly associated with the negative regulation of ROS overproduction and excess ROS are signaling molecules that trigger PCD in wheat seedlings during NaCl treatment. Our results suggest that *TaCDPK27* might play a positive role in conferring salt tolerance to plants.

## 4. Materials and Methods

### 4.1. Wheat Seedling Growth and Stress Conditions

Seeds of the wheat variety Henong 6425 (*Triticum aestivum* L.) were obtained from Tianjin Academy of Agricultural Sciences (China). The wheat seedlings were grown under hydroponic culture as previously described [45]. Two-leaf stage seedlings were selected and exposed to 0 mM NaCl, 150 mM NaCl, 5 mM LaCl_3_ (Sigma Aldrich, Saint Louis, MO, USA), and 5 mM LaCl_3_+150 mM NaCl for 6 days. Seedlings that received 0 mM NaCl treatment were used as controls.

### 4.2. Cloning, Sequencing, and Phylogenetic Analysis of TaCDPK27

Total RNA in the wheat root or leaf samples was extracted with TRIzol reagent (TaKaRa, Tokyo, Japan). Then, reverse transcription kits (Promega, Madison, WI, USA) were employed to reverse transcribe RNA into first-strand cDNA. From the salt stress-induced differentially expressed genes (DEGs) of wheat roots and leaves identified via an RNA-Seq assay, a unigene (accession no. *TraesCS5B02G109300*) released from EnsemblPlants was chosen and cloned [46]. Based on its CDS, the specific primers were designed to amplify the full length of ORF with reverse transcription-polymerase chain reaction (RT-PCR) (Table 3). The PCR experiment was conducted using TransStart Fast Pfu DNA polymerase (TransGen, Beijing, China). The PCR products were cloned with pGEM^®®^-T Easy (Promega, USA) and sequenced by Sangon Biotech (Shanghai, China). The molecular weight and theoretical pI were calculated with ExPASy software (http://www.expasy.org/, accessed on 1 December 2020). The BLASTp program (http://www.ncbi.nlm.nih.gov/, accessed on 1 December 2020) was employed to perform homologous comparison. BioEdit software (version 7.0.5.3) was used to perform multiple alignment. Smart software (http://smart.embl-heidelberg.de/smart/set_mode.cgi, accessed on 1 December 2020) was employed to predict the conserved domains and motifs. A phylogenetic tree was acquired using MEGA-X software with the neighbor-joining (NJ) method.

### 4.3. Subcellular Localization of TaCDPK27

Specific primers with XbaI and BamHI restriction sites were used to amplify the ORF of *TaCDPK27* without a termination codon from the first-strand cDNA or vector of *pGEM^®®^-T Easy-TaCDPK27*. The amplification products were digested with XbaI and BamHI, and were subcloned into the *pAN583:mCherry* vector at the N-terminus of the mCherry sequence using the pEASY^®®^-Basic Seamless Cloning and Assembly Kit (TransGen Biotech, China). Then, the *TaCDPK27-mCherry* fusion construct *pAN583:TaCDPK27-mCherry* was acquired. The *pAN583:TaCDPK27-mCherry* fusion construct and *pAN583:mCherry* (control) were transferred into wheat protoplasts, using the PEG-mediated transfection method as previously described [47]. The transformed protoplasts were cultured for 20 h at 25 °C. Then, the mCherry signals were examined with a confocal laser scanning microscope (Nikon Ti2 Eclipse A1, Nikon, Tokyo, Japan). The primers used are listed in Table 3.

### 4.4. BSMV-VIGS Assay

The BSMV-VIGS method was performed to acquire *TaCDPK27*-silenced wheat seedlings as previously described [48]. A cDNA fragment of 131 bp (+1612 bp to +1742 bp) was employed to acquire the *TaCDPK27*-silenced vector. After this fragment was inserted into the *BSMVγ* plasmid (BSMVγ:TaCDPK27), the plasmids *BSMVα*, *BSMVγ:GFP* and *BSMVγ:TaCDPK27* were linearized with *Mlu I* (Takara, Dalian, China). *BSMVβ* was linearized with *Spe I* (Takara, Dalian, China). Then, a RiboMAX large-scale RNA Production-T7 kit (Promega, Madison, WI, USA) was used to transcribe these linearized plasmids in vitro. Ribom 7G Cap Analog (Promega, Madison, WI, USA) was employed to produce the 5′-capped BSMV RNA molecules for subsequent BSMV-VIGS inoculation experiments. These experiments (including vector construction, in vitro transcription, BSMV-VIGS inoculation, *TaCDPK27*-silenced seedling identification, and silencing efficiency assessment of *TaCDPK27*) were conducted as previously described [45]. Then, the BSMV-VIGS-inoculated seedlings with three fully expanded leaves were treated with 150 mM NaCl for 6 days. All experiments had at least three separate replications. The primes used are listed in Table 3.

### 4.5. Physiological Measurements and Histochemical Staining

After 150 mM NaCl stress for 6 days, the following experiments were conducted. The in situ generation of H_2_O_2_ and O^2−^ in wheat leaves was examined using DAB and NBT staining, respectively. The staining procedures were carried out as previously described [45]. The quantitative determination of H_2_O_2_ and O^2−^ was performed following a previous study [46]. Evans blue staining was carried out to visualize the degree of cell damage in the wheat leaves under NaCl treatment. The leaf samples were immersed in 0.25% Evans blue staining solution and stained for 24 h in darkness. Then, the tissues were rinsed with decolorization buffer (absolute ethanol and glycerol, 9:1, *v*/*v*) until they became colorless. Subsequently, the leaves were photographed with a stereoscopic microscope (Nikon C-fled2). The activities of SOD, POD, and CAT, and the TUNEL assay were performed and analyzed as previously described [45].

The representative chlorophyll fluorescence parameters were chosen to test the PSII activity in wheat seedlings, according to our previous study [45]. The test parameters included the Fv/Fm, Y(II), qP, F0, ETR, and NPQ.

### 4.6. qRT-PCR Assay

Total RNA of the root and leaf samples was extracted with TRIzol reagent (TaKaRa, Tokyo, Japan). The extraction method, reverse transcription of the first-strand cDNA, and qRT–PCR were conducted as previously described [45]. The sequences of the primers used for qRT–PCR are listed in Table 3.

### 4.7. Cytosolic Ca^2+^ Fluorescence Measurement

The Ca^2+^-specific fluorescent probe Fluo-3/AM (Sigma, USA) was used to determine the level of Ca^2+^ in roots of wheat seedlings [45,49]. The 2–3 mm roots were loaded with 20 μM Fluo-3/AM for 2 h at 25 °C in the dark. Then the roots were washed 3–5 times with PBS to remove the excess Fluo-3/AM. The Ca^2+^ level in the roots was examined with a confocal laser scanning microscope (Nikon Ti2 Eclipse A1). The excitation wavelengths were 488 nm, and the emission wavelengths were in the range of 510–550 nm.

### 4.8. Statistical Analysis

Every experiment was performed with at least three repetitions. The data are presented as the mean ± SD. All statistical analyses were performed based on Duncan’s multiple range tests with SPSS software. *p* < 0.05 indicated significant differences.

## 5. Conclusions

A salt-responsive gene of wheat, *TaCDPK27*, was identified. The ORF length of *TaCDPK27* was 1875 bp, which encoded 624 amino acids with a predicted molecular weight and pI of 68.901 kDa and 5.6, respectively. TaCDPK27 belongs to subgroup III of the CDPK family of rice. The relative expression of *TaCDPK27* was significantly upregulated in the roots and leaves of wheat seedlings under 150 mM NaCl treatment. TaCDPK27 mainly exists in the cytoplasm. Under NaCl treatment, some of this protein was transferred to the membrane. Silencing of *TaCDPK27* in wheat seedlings significantly reduced their NaCl stress tolerance. The decrease in NaCl tolerance in *TaCDPK27*-silenced seedlings was due to excess ROS accumulation and increased levels of salt-induced PCD. *TaCDPK27* can function as a positive regulator of the salt stress response in wheat.

## Figures and Tables

**Table 1:** The effect of silencing *TaCDPK27* on growth of wheat seedlings under NaCl treatment.

Treatments	Root Length	Leaf Length
CK	36.50 ± 0.860 ^a^	31.10 ± 0.455 ^a^
γG	32.07 ± 0.262 ^b^	29.23 ± 0.249 ^b^
TaCDPK27	30.80 ± 0.170 ^c^	27.27 ± 0.287 ^c^
NaCl	29.00 ± 0.125 ^d^	25.97 ± 0.262 ^d^
γG+NaCl	25.77 ± 0.655 ^e^	24.33 ± 0.368 ^e^
TaCDPK27+NaCl	22.07 ± 0.478 ^f^	22.37 ± 0.340 ^f^

Note: The data are shown as mean ± SD (*n* = 3) of three independent experiments. CK was from wild-type wheat seedlings, γG was from BSMV-VIGS-GFP-inoculated wheat seedlings, and TaCDPK27 was from BSMV-VIGS-TaCDPK27-inoculated wheat seedlings. The data with different letters in same column show significant difference (*p* < 0.05).

**Table 2:** The effect of *TaCDPK27* silencing on chlorophyll fluorescence parameters of wheat seedlings under NaCl treatment.

Treatments	Fv/Fm	Y(II)	qP	F_0_	NPQ	ETR
CK	0.795 ± 0.002 ^a^	0.610 ± 0.002 ^a^	0.840 ± 0.003 ^a^	0.072 ± 0.000 ^a^	0.436 ± 0.101 ^f^	84.2 ± 0.309 ^a^
γG	0.788 ± 0.001 ^ab^	0.567 ± 0.001 ^b^	0.803 ± 0.002 ^b^	0.063 ± 0.004 ^b^	0.582 ± 0.016 ^e^	78.6 ± 1.717 ^b^
TaCDPK27	0.779 ± 0.001 ^b^	0.525 ± 0.002 ^c^	0.759 ± 0.006 ^c^	0.057 ± 0.002 ^c^	0.762 ± 0.004 ^d^	70.6 ± 0.818 ^c^
NaCl	0.757 ± 0.001 ^c^	0.464 ± 0.001 ^d^	0.736 ± 0.003 ^d^	0.048 ± 0.001 ^d^	0.976 ± 0.003 ^c^	65.2 ± 3.626 ^d^
γG+NaCl	0.737 ± 0.002 ^d^	0.364 ± 0.014 ^e^	0.605 ± 0.002 ^e^	0.040 ± 0.002 ^e^	1.137 ± 0.107 ^b^	50.4 ± 1.879 ^e^
TaCDPK27+NaCl	0.713 ± 0.016 ^e^	0.280 ± 0.043 ^f^	0.464 ± 0.004 ^f^	0.032 ± 0.001 ^f^	1.602 ± 0.052 ^a^	38.1 ± 1.306 ^f^

Note: The data are shown as mean ± SD (*n* = 3) of three independent experiments. CK was from wild type wheat seedlings, γG was from BSMV-VIGS-GFP-inoculated wheat seedlings, and TaCDPK27 was from BSMV-VIGS-TaCDPK27-inoculated wheat seedlings. The data with different letters in same column show significant difference (*p* < 0.05).

**Table 3:** Primers used in this paper.

Term	Gene	Primer Sequences (Forward/Reverse Primer)
ORF amplification	Calcium-dependent protein kinase 27 (TaCDPK27)	F:ATGGGCAACGTCTGCGT
		R:TTAGCTATTTGACTTTATGTTCAAGGCATCCCT
qRT-PCR	TaCDPK27	F:GCCGCCTTCCAATACTTT
		R:TTATCCTGATCTACTTCGCCTA
Subcellular localization	TaCDPK27	F: AAGTCCGGAGCTAGCTCTAGAATGGGCAACGTCTGCGT
		R: GCCCTTGCTCACCATGGATCCGCTATTTGACTTTATGTTCAAG
BMSV-VIGS	TaCDPK27	F: CAAACATTTTTTTTTTTTTTTAGCTAGCGCCGCCTTCCAATACTTT
		R:GATTCTTCTTCCGTTGCTAGCTTATCCTGATCTACTTCGCCTA

## Data Availability

Data are available from the authors on request.
